# Nitric Oxide Production and Regulation in the Teleost Cardiovascular System

**DOI:** 10.3390/antiox11050957

**Published:** 2022-05-12

**Authors:** Daniela Giordano, Cinzia Verde, Paola Corti

**Affiliations:** 1 Institute of Biosciences and BioResources (IBBR), National Research Council (CNR), Via Pietro Castellino 111, 80131 Napoli, Italy; daniela.giordano@ibbr.cnr.it (D.G.); cinzia.verde@ibbr.cnr.it (C.V.); 2 Department of Marine Biotechnology, Stazione Zoologica Anton Dohrn (SZN), Villa Comunale, 80121 Napoli, Italy; 3 Heart, Lung, Blood, and Vascular Medicine Institute, University of Pittsburgh, Pittsburgh, PA 15213, USA; 4 Division of Cardiology, Department of Medicine, University of Pittsburgh, Pittsburgh, PA 15213, USA

**Keywords:** nitric oxide synthase, Antarctic fish, hemoglobin, myoglobin, neuroglobin, cytoglobin, globin X, nitrite reductase, *S*-nitrosylation

## Abstract

Nitric Oxide (NO) is a free radical with numerous critical signaling roles in vertebrate physiology. Similar to mammals, in the teleost system the generation of sufficient amounts of NO is critical for the physiological function of the cardiovascular system. At the same time, NO amounts are strictly controlled and kept within basal levels to protect cells from NO toxicity. Changes in oxygen tension highly influence NO bioavailability and can modulate the mechanisms involved in maintaining the NO balance. While NO production and signaling appears to have general similarities with mammalian systems, the wide range of environmental adaptations made by fish, particularly with regards to differing oxygen availabilities in aquatic habitats, creates a foundation for a variety of in vivo models characterized by different implications of NO production and signaling. In this review, we present the biology of NO in the teleost cardiovascular system and summarize the mechanisms of NO production and signaling with a special emphasis on the role of globin proteins in NO metabolism.

## 1. Introduction

The history of nitric oxide (NO) in biology dates back to the origin of life. In the Earth’s ancestral atmosphere, the formation of NO may have been a critical defense mechanism against the oxidative destruction by toxic levels of ozone [1]. Later, organisms developed other biological functions for NO formation. Taking advantage of NO toxicity to viruses and parasites, some organisms directed this ubiquitous pathogen-killing mechanism available in nature for their own immune defense [2]. In addition, NO has been incorporated in a number of cellular mechanisms and it is involved in several physiological and pathological processes. The nature of NO as a signaling molecule was revealed through extensive scientific research on mammalian models where NO was identified in the endothelium as the endothelium-derived relaxing factor, a key regulator of vasodilation. This discovery led to the awarding of the Nobel Prize in 1998 [3,4,5,6]. Since then, the initial knowledge of NO function in host-defense mechanisms against microbial pathogens has been largely extended and many studies have identified NO as a crucial factor, not only in endothelial signaling, but including and not limited to neuronal signaling and immune response.

**Figure 1:**
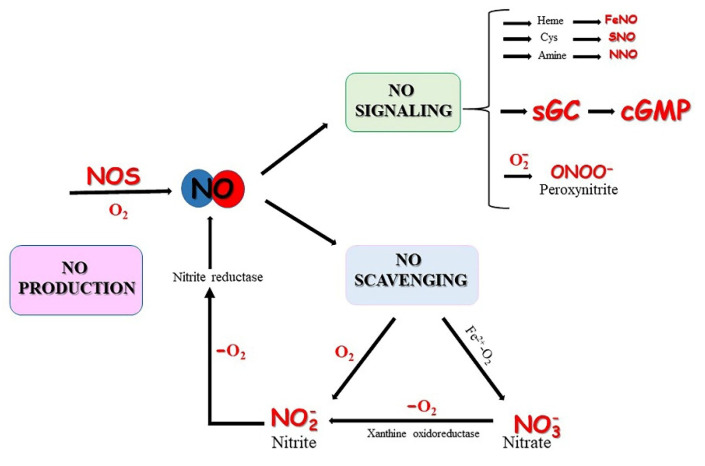
Production and fate of NO. The major enzymatic pathway for NO synthesis is catalyzed by NOS isoforms in the presence of O_2_. Once produced, NO can be rapidly converted to nitrite with dissolved O_2_ by NO scavenging reactions, or can be inactivated to nitrate by oxygenated heme proteins. When *de novo* production via NOS is compromised under low O_2_, NO can be produced by the reduction nitrite in heme proteins, or by nitrate conversion to nitrite by xanthine oxidoreductase. NO signaling occurs by binding to (i) sGC promoting the canonical cGMP cascade, or (ii) heme-containing proteins, thiols, or amines, forming iron-nitrosyl (FeNO), S-nitroso (SNO) and *N*-nitroso (NNO) compounds promoting protein post-translational modifications. Excess NO can react with superoxide to form peroxynitrite (ONOO^−^), which in turn can react with lipids, DNA, protein thiols, and oxidize cysteine residues.

NO is implicated in a number of major cellular functions including cell proliferation, differentiation, apoptosis, macrophage activity, and neurotransmission [7,8]. NO production is achieved through non-enzymatic and enzymatic reactions, which can coexist within the same cell or tissue [9]. In the presence of oxygen (O_2_) and at a physiological pH, NO is mainly produced enzymatically by NO synthases (NOSs) from *_L_*-arginine [10]. Conversely, in low O_2_ conditions NO production by NOS enzymes is inefficient and other systems are involved. NO can be generated from nitrite by non-enzymatic or catalytic reduction, in which heme- and molybdopterin-containing proteins can assume important roles as NO producers [11,12]. As such, nitrate and nitrite serve as inert bioavailable reservoirs in the blood and tissues, where they can be converted into NO during hypoxia by heme proteins playing an enzymatic role as nitrite reductases [13,14,15], (Figure 1). At levels within the picomolar to nanomolar range, NO activates its target soluble guanylate cyclase (sGC), which promotes the generation of cyclic guanosine 3’,5’-monophosphate (cGMP) [16,17], critical for the regulation of blood flow and vasodilation [18]. At higher concentrations, NO can react with O_2_ radicals generating nitrogen radical species that lead to oxidative stress with negative consequences for living cells [19,20,21], (Figure 1).

Similarly to mammals, in fish NO mediates fundamental signaling pathways. Although reports on NO metabolism and signaling in fish still remain relatively uncommon compared to mammalian systems, various studies have shown that fish tissues can generate NO [22] and utilize it for signaling and as a defense mechanism [23,24]. Teleost species consist of about one half of all the vertebrate species known on Earth today and have conquered nearly all of the planet’s aquatic habitats. Temperature and O_2_ availability have been the major drivers in the evolution of fish. The long evolutionary history, the different O_2_ requirements between species, and different adaptation responses to environmental conditions make fish excellent models for the study of O_2_-dependent molecular and cellular mechanisms and functions. Notably, NO metabolism and signaling are highly dependent on environmental O_2_ and O_2_ concentrations in water are highly dependent on temperature, currents, and salinity. Fish have adapted to live within diverse levels of O_2_ saturation, ranging from the nearly anoxic deep sea to high altitude lakes, and from the poorly oxygenated pools of warm desert springs to the highly oxygenated frigid waters of the Southern Ocean. For instance, the crucian carp (*Carassius carassius*) and goldfish (*C. auratus*) have evolved to tolerate prolonged and severe hypoxia conditions [25] while other species, including trout (*Oncorhynchus mykiss*), are less tolerant to even short hypoxic episodes [26]. In this context, zebrafish (*Danio rerio*), being characterized by a growth-dependent transition from hypoxia tolerance to sensitivity [27], is widely accepted as an adequate model system not only to study human diseases [28], but also to study the mechanisms of environmental acclimation to hypoxia [29].

Heme globins are important regulators of NO homeostasis in mammals and also in fish they are central regulators of NO physiology. While all teleosts exhibit a remarkable multiplicity of hemoglobin (Hb) in their blood with different functional properties for adapting to changing environments, red-blooded notothenioid species inhabiting the frigid waters of the Southern Ocean show a phylogenetic trend toward decreased Hb multiplicity (reviewed in [30]). Icefish, the modern family of Notothenioidei, are the only species of vertebrates able to survive without Hb and to have evolved in the absence of red blood cells. The loss of erythrocytes was driven by environmental changes over evolution which led to a decrease in temperature of the Southern Ocean waters and a dramatic increase in dissolved O_2_ concentration [30]. Myoglobin (Mb) expression is also absent from the hearts of 6 out of 16 icefish species [31,32], but all the species appear to retain the other globins such as neuroglobin (Ngb), cytoglobin-1 (Cygb-1), cytoglobin-2 (Cygb-2), and globin X (GbX) [33,34,35,36,37]. Icefish are often proposed as a model organism for studying the up-regulation of endothelial NO signaling because of their lack of Hbs and Mb [38]. The cardiovascular adaptations (e.g., large hearts, expanded vascular, capillary networks, blood volume, and cutaneous respiration) compensate for the loss of an O_2_ carrier and are thought to be linked to NO signaling [31,39,40]. In addition, the cardiac muscle in myoglobinless icefish is characterized by an increased number of mitochondria to augment aerobic power and/or facilitate O_2_ diffusion [41]. The homeostatic activity of NO probably facilitated the evolution of these compensatory traits “jump-started”, as suggested by Sidell and O’Brien [31]. 

In this review, we discuss the pathways of NO formation and the mechanisms of NO signaling in the cardiovascular system of teleosts through the analysis of different fish adapted to different aquatic environments with a special emphasis on the icefish from the Southern Ocean. Similarly to mammals, in fish NO is mainly produced by NOSs enzymes, but in conditions of low O_2_, other mechanisms are activated in which nitrogen compounds and globin proteins modulate NO levels to finely regulate NO homeostasis. In the cardiovascular system of teleosts, NO is shown to signal through the canonical NO-sGC-cGMP signaling axis. However, NO signaling through post-translational modifications of globins is particularly relevant in fish as a consequence of their unique capacity among vertebrates to adapt to different aquatic environments.

## 2. Oxygen-Dependent NO Synthesis

The major enzymatic pathway for NO synthesis consists of a reaction catalyzed by a family of NOS isoforms (EC 1.14.13.39) featuring the oxidation of the guanidino group of *_L_*-arginine with molecular O_2_ to produce *_L_*-citrulline and NO [10]. In mammals, there are three different NOS isoforms: neuronal NOS1 (nNOS), NOS2 or inducible NOS (iNOS), and NOS3 or endothelial NOS (eNOS) encoded by three distinct Nos genes ([42] and reference therein). Nos genes were named according to the tissues where they were first identified: nNos in neurons, iNos in immune-activated macrophage cell lines, and eNos in the vascular endothelium. However, later they have been found to be widespread in a variety of tissues and cell types [43]. nNOS has been found constitutively expressed, mainly in central and peripheral nervous systems, regulating blood pressure, smooth muscle relaxation, and vasodilation. iNOS has been found in many cell types induced by lipopolysaccharides, bacterial products, and cytokines during inflammatory diseases and septic shock, whereas eNOS is mostly expressed in endothelial cells, controlling the dilation of blood vessels and blood pressure [7]. An additional NOS isoforms, a nNOS homologue has been identified in the matrix and inner membrane of mitochondria (mtNOS), where it is involved in the regulation of O_2_ and the biogenesis of mitochondria [44,45,46,47].

The modulatory mechanisms of NO signaling in vertebrates revolves around NOS interactions with cofactors and Ca^2+^ availability, post-transductional modifications, and protein–protein interactions [48], and these mechanisms are largely dependent on the subcellular localization of NOS isoforms [49]. The constitutive isoforms nNOS and eNOS are Ca^2+^-dependent due to the presence of an inhibitory loop in the subdomain of flavin mononucleotide (FMN) of which they are constituted, while the inducible isoform (iNOS) is Ca^2+^-independent due to the lack of this loop [50,51]. Contrary to nNOS and eNOS, directly signaling with proximal targets through NO production, iNOS is initially expressed in the cytosol and is then recruited to phagosomes or peroxisomes where elevated NO produced by iNOS reacts with superoxide to form peroxynitrite involved in the host’s defense against pathogens [52,53].

All NOSs are homodimers with each monomer consisting of a carboxyl-terminal reductase domain and an amino-terminal oxygenase domain, both linked by a calmodulin (CaM) site. The reaction requires nicotinamide adenine dinucleotide phosphate (NADPH), FMN, and flavin adenine dinucleotide (FAD) as cofactors all responsible for the serial transfer of electrons to the heme of the oxygenase domain (6R)-5,6,7,8-tetrahydrobiopterin (BH_4_) [7,54,55]. The reduced heme of NOS then binds O_2_ which oxidizes the guanidine group of the *_L_*-arginine to produce *_L_*-citrulline and NO.

### Evolution and Expression of NOSs

The evolutionary events originating in the different NOS isoforms in animals are still under debate. NOS-like enzymes were found in bacteria [56,57], but independent events gave rise to the *Nos* genes in prokaryotic and eukaryotic lineages [58]. Metazoan NOS isoforms display a highly-conserved protein sequence and gene organization in terms of their intron position and phases, pointing to the importance of their function in almost all lineages. However, important genome duplication events occurred in numerous lineages, leading to the independent acquisition of novel functions through gains and losses of protein motifs [42]. In vertebrates, an ancestral *Nos* gene was duplicated generating *Nos1* and *Nos2* after the agnathan–gnathostome split; during a second duplication event in which tetrapod evolution occurred, *Nos3* was generated from *Nos1*, leading to the three isoforms present in current mammals [42].

**Figure 2:**
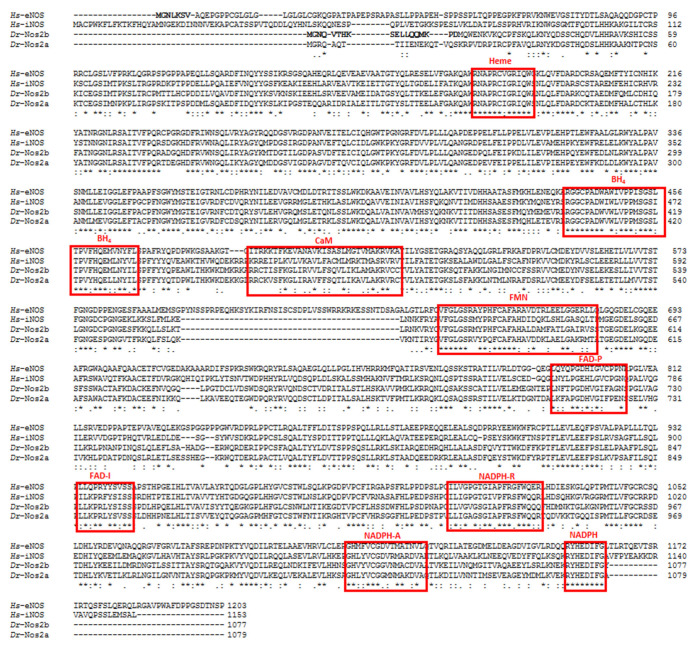
Alignment of the deduced amino acid sequences of human eNOS (*Hs*-eNOS, accession number M93718), human iNOS (*Hs*-iNOS, accession number L09210.1), zebrafish Nos2a (*Dr*-Nos2a, accession number AM749801.1), and Nos2b (*Dr*-Nos2b, accession number AM749802.1). The sequences were aligned using ClustalO. Identical amino acids are indicated by asterisks, conservative substitutions are shown by a colon, and semi-conservative substitutions by dots. The predicted *N*-myristoylation site at *N*-terminals are in bold; the conserved cofactor-binding sites for heme, BH_4_, CaM, FMN, FAD pyrophosphate (FAD-P), FAD isoalloxazine (FAD-I), NADPH ribose (NADPH-R), NADPH adenine (NADPH-A), and NADPH binding are boxed in red. Conserved binding sites are taken from [59,61].

Following the fish–tetrapod split, *nos* and *nos2* genes were duplicated again in the teleost lineages during a third duplication event, but through later losses only two genes (*nos1* and *nos2*) have been maintained: *nos1* is present as a single copy, whereas *nos2* in some teleost duplicated into different forms [42,59]. *nos2* was subjected to a complex evolutionary scenario in teleost because of the additional fish specific gene duplication, resulting in some cases in the presence of different *nos2* paralogs [60]. The Zebrafish genome encodes two *nos2*, *nos2a* and *nos2b*, the former is inducible and found in the spleen, kidney, muscle, gut, ovary, and skin but not in the heart, liver, and testis, whereas the latter is constitutively expressed and widespread in all these organs, including the heart, liver and testis [59]. Zebrafish *nos2* represents an example of gene divergence where *nos2b* has an orthologous position with mammalian *eNos* and exhibits similar functions, whereas Nos2a is encoded by *nos2a* mapped on a different chromosome with respect to *nos2b* and functions like the mammalian iNOS. Indeed, Nos2b displays a myristoylation consensus sequence at the *N*-terminus characteristic of mammalian eNOS, thus suggesting functional homologies with the mammalian enzyme [59], (Figure 2*).* However, a comparison of the amino acid sequences of Nos2a and Nos2b with human eNOS and iNOS displays a similar sequence identity between them. Indeed, both Nos2a and Nos2b share 51% of sequence identity with eNOS and 58% identity with iNOS.

To date, *nos2* homologs have been also identified in the goldfish *C. auratus* [62], rainbow trout *O. mykiss* [63], carp *Cyprinus carpio* [64], Atlantic salmon, *Salmo salar* [65], pacu *Piaractus mesopotamicus* [66], and channel catfish *Ictalurus punctatus* [67]. In the goldfish and carp, three genes *nos2a*, *nos2ba*, and *nos2bb* have been found; in *S. salar* and *O. mykiss*, two different copies of *nos2*, *nos2*α, and *nos2β* have been identified; *nos2.1* and *nos2.2* have been found in the channel catfish *I. punctatus* [60]. *nos2* homologs are lacking in the genomes of fugu *Takifugu poecilonotus*, tetraodon *Tetraodon nigroviridis*, stickleback *Gasterosteus aculeatus*, and the medaka *Oryzias latipes* species [59]. 

It appears that none of the teleost genomes annotated to date present the *nos*3 gene, and there is no molecular evidence for endothelial *nos* in fish [42,59,68,69,70]. The identification of a *nos3* ortholog in the ray-finned fish spotted gar *Lepisosteus oculatus*, a holostean fish (the sister group of teleost), changed the evolutionary perspective of *nos* [68]. Recently, Annona et al. [60] reported that the identification of *nos3* genes in the genomes of ray-finned fish of non-teleost lineages and in the teleost elephantfish *Paramormyrops kingsleyae*, belonged to their osteoglossomorph lineage. No *nos3* gene had been identified in the other lineages of teleosts. However, the identification of a *nos3* ortholog in the catshark *Scyliorhinus torazame* suggests its presence in the ancestor of gnathostomes. Studies in fish report a significant level of responsiveness by the endothelial smooth muscles to the application of NO and NO donors causing significant vasodilation [71], thus raising the question about the existence of a different possible source of NO in the vasculature.

Although no *nos3* gene had been identified in the majority of teleosts [60,72], other Nos isoforms seem to have acquired this function. Data obtained using mammalian anti-eNOS antibodies demonstrated the presence of an endocardial-endothelial NO source involved in cardiac modulation in the teleost species *Anguilla anguilla*, *Thunnus thynnus thynnus*, and *C. auratus*, and in the icefish *Chionodraco hamatus* and red-blooded *Trematomus bernacchii* [73,74]. An eNOS-like enzyme was also found expressed in the lungs, gills, kidneys, heart, and skeletal muscles of the non-teleost lungfish species *Protopterus dolloi* and *P. annectens* [75,76,77], and Nos1 from agnathan *Petromyzon marinus* possesses an endothelial-like consensus typical of eNOS [42]. After three days post-fertilization (dpf), zebrafish larvae the cardiomyocytes showed immunoreactivity to the antibody used for mammalian eNOS and, in response to the NO donor sodium nitropusside (SNP) and the NOS inhibitor nitro-*_L_*-arginine methyl ester (*_L_*-NAME), the main axial vessels react with a significant change in the vessel diameter. Nos2b appears to be the main cause of vasodilation in zebrafish larvae [78]. Nos1 may be most likely the source of NO in perivascular nitrergic neurons that innervate the vasculature of some teleost species [68,71]. The presence of *nos* genes in different species is summarized in Table 1.

The expression (*C. hamatus* and *T. bernacchii*, [73]) and function (icefish *C. hamatus*, [80]; icefish *Chaenocephalus aceratus* and red-blooded *T. bernacchii*, [81]) of Nos have been demonstrated also in Antarctic fish hearts. In addition to the high level of Nos1, constitutively expressed in five icefish species [82], Nos3 and Nos2 have been found in the heart of red-blooded and icefish species [81]. In the heart, total Nos activity can be almost totally addressed to Nos3, mainly localized in the atrial and ventricular endocardium and epicardium and, to a lesser extent, in the myocardial trabeculae [81]. Nos3 may be particularly important in the icefish for protecting its myocardium, ensuring adequate O_2_ transcellular transport from the lacunae to the mitochondria, and contributing to antithrombotic homeostasis in the heart ventricle [83]. Nos2 is exclusively present in the cardiomyocytes and in the epicardium of icefish. However, *C. aceratus* (Hb^−^/Mb^−^) shows a lower cardiac basal expression of Nos3 and Nos2 with respect to *C. hamatus* (Hb^−^/Mb^+^) [73]. The absence of Mb in *C. aceratus* may lead to a reduction of Nos expression as a consequence of the greater availability of free NO. The cytoplasmic expression of Nos2 in cardiomyocytes may regulate both mitochondrial respiration through cytochrome c oxidase inhibition [84] and myocardial contractility [85].

Nos expression, and thus its activity, can also be modulated by O_2_ availability and temperature changes. Interestingly, under heat stress the expression of Nos3 and Nos1 is enhanced in the gills of *C. hamatus*, whereas it is reduced or disappeared in *T. bernacchii*, suggesting a specie-specific morpho-functional response of the two Antarctic teleost to heat stress [83,86]. In *A. anguilla* hearts, NOS activity is impaired when the eel is exposed to temperatures lower or higher than the acclimation ones and this is associated with the reduced expression levels of phosphorylated forms of Nos3 and protein kinase B (Akt) [87]. In the Atlantic salmon, long-term exposures to high temperature is characterized by an increased expression of Nos2 in myocardium with an increase of vascular endothelial growth factor (VEGF) expression, suggesting that temperature stress accounts for an increasing vascularization associated with vasodilation (via NO) in order to increase the blood flow to the heart [88].

In fish and similar to mammals, the transcription of iNOS and eNOS is under the control of the hypoxia-inducible transcription factor HIF-1α [89,90,91]. This may explain the increased myocardial Nos3 expression associated with the high expression of HIF-1α in the goldfish heart exposed to hypoxia [79]. Similarly to mammalian models [92], in the perfused goldfish heart, hypoxia activates NOS-dependent NO production mediated by the PI3-K/Akt kinase pathway, which is protective for the heart. An increased NOS expression in response to hypoxia has been also observed in the vasculature of the trout [93].

## 3. Nitrite-Dependent NO Formation

Nitrate and nitrite have previously been considered inert compounds derived from the oxidation of NO without any metabolic function in the circulation. However, it has become increasingly appreciated that nitrite and nitrate are important sources of NO upon enzymatic reduction. This process is particularly relevant in hypoxia as low O_2_ levels compromise NOSs activity [18,94,95,96,97,98]. The oxidative pathway (NOS) and the reductive pathway (NO_2_^−^) of NO production can work synergistically in vivo to maintain NO levels in response to changes in O_2_ tension. A number of proteins involved in oxidative processes at physiological O_2_ conditions can become reductive enzymes as O_2_ is depleted and can catalyze the reduction of nitro compounds to release NO. O_2_ levels can impact the oxidation/reduction properties of heme- and molybdopterin-containing proteins such as the globins and xanthine oxidase, favoring heme—electron transfer reactions, such as the reduction of nitrite to generate NO (Equation (1) [11,99,100,101]. The presence of these nitrite reductases in various compartments of the vasculature and cardiac tissue makes them central actors in the regulation of NO levels in vascular biology.

Fe^2+^NO_2_^−^ + H^+^→Fe^3+^ + NO + OH^−^ (1)

It should be noted that competing reactions, namely the scavenging of NO by the ferrous heme, greatly decrease the amount of NO produced by NOS. In presence of O_2_ in the environment, the Fe^2+^ bound to O_2_ converts NO to nitrate according to Equation (2) named NO dioxygenation.

Fe^2+^O_2_ + NO→Fe^3+^ + NO_3_^−^ (2)

**Figure 3:**
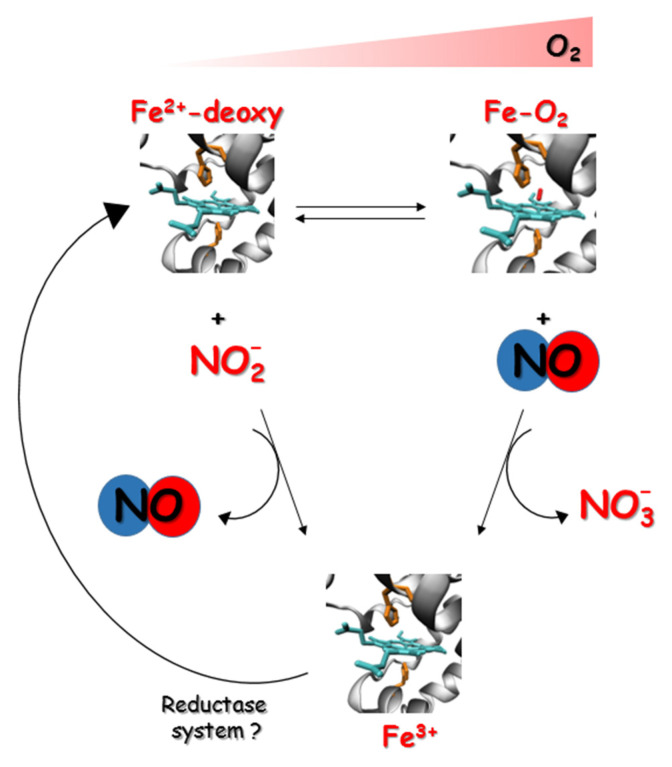
NO homeostasis in O_2_ gradient. Globins function as NO scavengers by oxidizing NO to nitrate or as NO producers by reducing nitrite to NO based on O_2_ concentrations. The presence of the reductase system may allow the reduction of ferric to ferrous heme.

Globins, as heme centers, may function as NO scavengers or NO producers based on O_2_ concentrations (Figure 3).

In fish, the role of globins in producing and scavenging NO is well-documented in the vasculature and heart and will be discussed later on in this study. Since fish are exposed to an additional direct uptake of exogenous nitrite from the aquatic environment across the gills as opposed to terrestrial animals [102], a low concentration of nitrite in water is an important source of NO at a low O_2_ tension. In fact, nitrite exerts important biological functions at low concentrations [14] but it is toxic at high concentrations, particularly in fish [103]. An additional source of nitrite in tissues is nitrate. While mammalian tissues have been shown to be able to reduce nitrate to nitrite—a process mediated by xanthine oxidoreductase and possibly other proteins—in fish this still remains to be established, but it could be an important source of nitrite and thus NO in hypoxic tissues. In the crucian carp, in conditions of severe hypoxia, nitrite uptake through the gills from the environment reaches the heart rapidly [104], where nitrite surplus may be correlated to Mb exerting a nitrate reductase activity [105]. In hypoxia-tolerant fish such as the goldfish, basal nitrite levels in the plasma are similar (0.75 μM) to those observed in mammals (about 0.1–0.8 μM). Similar values have been observed in the hypoxia intolerant fish flounder, eelpout, oyster toadfish, and brown trout (about 0.2 μM) [102,106], suggesting that when ambient nitrite is sufficiently low, the nitrite plasma level reflects the constitutive NOS activity comparable in all teleost species. 

In fish hearts, the major target of hypoxia, a reduced O_2_ availability determines an increased NOS expression or, alternatively, a nitrite reduction in NO to stabilize NO levels, thus protecting the myocardium from hypoxia [79,106,107,108]. In the heart of the Antarctic *C. hamatus* and *T. bernacchii*, nitrite influences cardiac performance by inducing a concentration-dependent increase of contractility, and its conversion to NO requires the nitrite reductase activity of xanthine oxidase and cytochrome P-450 [109,110]. For the first time in Rochon et al., nitrite has been associated with cardiac regeneration. In hypoxic conditions, exposure to physiological levels of nitrite can improve the heart’s ability to regenerate in zebrafish amputation and cryoinjury models. Nitrite modulates the initial immune response by stimulating neutrophils and macrophage migration to the site of injury, resulting in an anticipation of the proliferative events taking place in the cardiac muscle and ultimately reducing the injury size during the early regeneration process [111].

### 3.1. NO Formation by Globins

Over the past two decades, studies of the genetics and function of globin proteins have opened up new paradigms for the role of globins in NO biology. The ubiquitous expression of vertebrate globins in tissues and their biochemical properties supports a central function in NO metabolism and signaling [112]. It is now well-documented in mammals that in vivo Hb in the blood and Mb in the heart act as nitrite reductases, and the production of NO from nitrite induces vasodilation in the vasculature and is cytoprotective for the heart [97,113,114,115,116,117]. In the microcirculation of fish where O_2_ tensions are lower than mammalian systems and that do not specifically express endothelial NOS, the reduction of nitrite mediated by globins may be an essential source of NO. Biochemical studies have also demonstrated that human Ngb and cytoglobin (Cygb) display nitrite reductase activity mainly dependent on the formation of an intramolecular disulfide bridge [118,119]. Moreover, the discovery of additional globins in the red blood cells of fish [120,121], and the documented nitrite reductase activity of zebrafish Cygb-1 [121] and GbX in the zebrafish blood [96], support the central role of globins in NO homeostasis in the vasculature in fish as well. In vitro studies have identified a significant globins homology between humans and fish with respect to the mechanisms involved in globins reduction. Similarly to mammals, the zebrafish Cygbs are shown to be reduced by the cytochrome b5/cytochrome b5 reductase system, allowing for its continuous catalytic oxidoreductase activity [122,123].

Globins typically contain ~150 amino acids organized in eight α-helical segments (named A through H) folded in a 3/3 α-helical sandwich structure that surrounds the heme group. Globin functions are centered on the heme, the main ligand-binding site in these proteins. Vertebrates have evolved a complex superfamily of globins, including not only the well-known tetrameric Hb (α_2_β_2_) in red blood cells and monomeric Mb in muscles, but also androglobin (Adgb) [124], Cygb [125], globin E (GbE) [126], globin Y (GbY) [127], GbX [128], and Ngb [129]. Remarkably, only three vertebrate species express all eight globins: the “living fossil” *Latimeria chalumnae*, a member of the coelacanth order of lobe-finned fishes, and closely related to lung fishes, the Chinese soft-shell turtle (*Pelodiscus sinensis*), and the Western painted turtle (*Chrysemys picta bellii*). Likely, the different environmental conditions and the life—history traits have influenced their presence or absence. After whole and local genome duplication events, the vertebrate globin gene repertoire has evolved to provide different expression patterns and tissue-specific roles [130].

**Figure 4:**
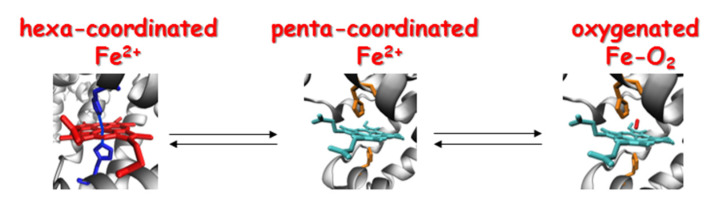
The shift from hexa-coordinated to penta-coordinated heme depends on the iron reactivity and affinity with the distal internal ligand.

In the deoxygenated state, Hb, Mb, and GbE are penta-coordinated, with the sixth coordination site of the ferrous iron unbound and therefore free to bind potential ligands. Their most prominent role is to transport and store O_2_ for oxidative metabolism. In contrast, Ngb, GbX, Adgb, and Cygb are hexa-coordinated with the distal histidine bound to the sixth coordination site of the iron heme in the deoxy state. These globins are more prone to electron transfer reactions rather than ligand binding, suggesting novel non-respiratory functions ([112] and references within). The equilibrium between penta-coordinated and hexa-coordinated heme depends on the binding affinity of the heme with the distal histidine of the polypeptide chain and the competition with exogenous ligands. These characteristics highly influence the reactivity of the heme proteins and their function [131], (Figure 4).

Research over the past two decades yielded a precise and complete picture of the structure of hexa-coordinated states [132,133], although the physiological functions of these proteins still remain elusive. Endogenous hexa-coordination was also found in the Antarctic teleosts Hbs [134], Ngb [36], and Cygbs [33,34,35], suggesting a possible involvement of these globins in the catalytic mechanisms of electron transfer, with the hexa-coordinated states acting as intermediates prone to easy reduction or oxidation. The blood Hbs of Antarctic fish, characterized by β chains with a strong propensity to form hexa-coordinated bis-histidine adducts [135], adopt peculiar oxidation states under native-like conditions suggesting a potential involvement of these proteins in functional redox processes yet to be identified and/or in scavenging reactive oxygen species (ROS) [134].

In mammals as well as in fish, the reaction of Hb and nitrite is strongly dependent on the allosteric equilibrium between the tensed (low O_2_ affinity) and relaxed state (high O_2_ affinity) of the Hb. Hbs with a high O_2_ affinity (i.e., allosteric equilibrium shifted towards the R state) are faster nitrite reductases than Hbs with a low O_2_ affinity. These assumptions thus suggest that hypoxia-tolerant fish (e.g., carp and crucian carp), which have evolved Hbs endowed with very high O_2_ affinity [136], are more prone to promote NO production through nitrite reduction compared to hypoxia-intolerant species [102,137]. Indeed, the Hbs of carp and crucian carp [138] have faster nitrite reductase activity than the Hbs of rainbow trout and brown trout with a low O_2_ affinity, and are predicted to be less relevant in NO production mediated by nitrite reduction [137].

In addition to the main role of storage and supply functions, Mb may also be involved in the regulation of NO and ROS levels in response to O_2_ homeostasis. Since Mbs arose very early in evolution when simple organisms did not yet have the need to develop an O_2_ storage system, it is possible that their ancestral roles were related to the metabolism of NO and other gaseous ligands [139]. In teleost, Mb is found in skeletal and heart muscle in addition to a variety of tissues including the endothelial cells, brain, and liver where it can exist in more than one isoform. In some cyprinid fish like the common carp (*C. carpio*) and the goldfish *C. auratus*, the *mb* gene duplicated within the lineage into *mb1* and *mb2*. *mb1* occurs in oxidative muscle and it is also ubiquitously expressed in several other tissues, whereas *mb2* is specific to the brain [140,141]. The two isoforms possess quite different functions, which may account for some part of the tetraploid cyprinid fish hypoxia tolerance [140]. In these hypoxia-tolerant fishes, NO is supplied in part by the Nos activity, but the reduction of nitrite in the heart seems to be the predominant mechanism strongly dependent on the extent of Mb deoxygenation and O_2_ binding affinity. In fact, in the anoxic goldfish heart, Mb with a high O_2_ binding affinity strongly contributes to hypoxia tolerance and during severe hypoxia exhibits high nitrite reductase activity rates, whereas in trout, the presence of Mb with low-O_2_ binding affinity appears to be mainly functional for O_2_ diffusion at high O_2_ tensions [26].

Zebrafish Mb displays the same tissue distribution and microvascular location as in carp [142]. Interestingly, in the gills of carp and zebrafish the Pillar cells express a high level of Mb [142], suggesting a possible role for Mb in the regulation of blood pressure in gills. These results are in line with the proposed function of Mb in NO metabolism [143] and with the hypothesis that NO is strongly involved in vascular control [144,145]. The up-regulation of *mb* expression in the icefish *C. hamatus* under hypoxic stress is of particular interest [33]. *C. hamatus* is one of the few icefish species that has retained the Mb function [31,40], suggesting that *mb* may preserve a protective role in the icefish during hypoxia. Likewise, in red-blooded fish the up-regulation of *mb* mainly in the brain and gills after hypoxic stress, indicates that this globin may contribute to the tolerance of low-O_2_ levels, as what similarly occurs in goldfish [141], and could be either related to ROS scavenging [142] or maintaining a NO balance [117,146].

### 3.2. NO Scavenging by Globins

Once produced, free NO is a radical species highly that is reactive with other O_2_ radicals in forming reactive nitrogen species (RNS). While it is now appreciated that small amounts of RNS can induce signal transduction in the cell through *S*-nitrosylation, high RNS can react with DNA, protein thiols, and cysteine residues to alter the catalytic activity of enzymes and ultimately induce cell death. It is imperative for the cell to keep NO levels under a tight control with multiple mechanisms in place to finely tune NO amounts depending on the needs of the cell. The biological importance of globins in regulating NO balance and the O_2_-dependent redox reactions involved in the metabolism of NO [23] are particularly relevant in fish, adapted to the most diverse aquatic habitats ranging from nearly anoxic waters to the oxygenated and frigid waters of the Southern Ocean.

Perhaps the most interesting evidence of NO scavenging by globin proteins in fish comes from a number of studies conducted regarding the Antarctic fish. In these fishes, the total concentration of NO metabolites in plasma is higher in icefishes than in red-blooded notothenioids [e.g., *C. aceratus* (Hb^−^/Mb^−^), 22.7 ± 2.9 μM; *Notothenia coriiceps* (Hb^+^/Mb^+^), 14.7 ± 1.7 μM], suggesting a higher NO load in icefish. High NO levels do not appear to be a result of higher NO synthesis but rather of its decreased degradation due to the absence of the NO-scavenger Hb [147]. All icefish have low O_2_ demands and have evolved in habitats where O_2_ has been constantly saturated. Considering the role of Mb and Hb in supporting tissue performance, Mb and Hb deficit likely affects the ability to meet tissue O_2_ demands in unstable conditions and to adapt to environmental changes [39]. The experimentally-induced anemia of the red-blooded notothenioid *N. coriiceps* with the hemolytic agent phenylhydrazine (PHZ) produced an increase in the circulation of NO. On note, there is no increase in Nos activity in the tissues of PHZ-treated *N. coriiceps* or of *C. aceratus*. This increase appears to be associated with the reduction in functional Hb and consequent absence of NO scavenging in the blood [31].

High levels of Mb expression are generally associated with lifestyles or environments that demand efficient O_2_ delivery. For example, high levels of Mb are present in the muscles of diving mammals and birds [148]. Conversely, the selection of high Mb levels may be relaxed when needs for O_2_ delivery are low as demonstrated in some icefish lineages that independently lost Mb expression, following the earlier loss of Hb [31]. High NO levels occurring in icefish in the absence of both Hb and Mb have promoted some of the major cardiovascular and subcellular compensations (i.e., large hearts, large vascular and capillary networks, and blood volume) [31].

Cygb is ubiquitously expressed in vertebrate tissues and it has been proposed to be involved in various physiological processes: (i) O_2_ supply to mitochondria [125,149,150], (ii) NO scavenging through deoxygenation [151], (iii) NO production by nitrite reduction under anaerobic conditions [152], (iv) the regulation of NO levels and metabolism in the vascular walls [153], and (v) protection against RSN and ROS [154]. In teleosts, two distinct paralogous genes, *cygb-1* and *cygb-2*, are present. The sequence alignment of human and fish Cygbs indicates that *cygb-2* is more closely related to mammalian Cygb than *cygb-1* [128]. Although both Cygb mRNAs find broad expression in many tissues, high levels of *cygb*-2 were detected in the brain and retina of zebrafish [155].

Similar to other vertebrate Cygbs [121,132], Antarctic fish Cygb-1 and Cygb-2 present a hexa-coordinated heme group [33,34,35] with a high-O_2_ affinity, unlikely supporting an O_2_-delivery role. Antarctic Cygbs-1 exhibit a slow rate for nitrite reductase activity and do not catalyze peroxynitrite isomerization [34]. Similar to Cygbs, the hexa-coordinated Antarctic Ngbs, showing an autoxidation rate higher than those typical for O_2_-transport proteins, do not appear to function as O_2_ carriers [36]. Antarctic Cygbs-1 and Ngbs often display large internal cavities, potential tunnels, and gates in their structure. These features of the hexa-coordinated globins may assist multi-substrate reactions such as the NO dioxygenase, by providing a close-by reservoir of secondary reactants and sustaining catalytic turnover. The enlarged cavities found in these icefish globins may be important in sequestering the excess circulating NO levels, thus prompting the need to compensate for the loss of Hb and Mb scavengers [34,36].

Surprisingly, in zebrafish Cygb-1 was recently found in the penta-coordinated state, whereas Cygb-2 exhibited hexa-coordination [121]. The slower autoxidation rate and fast rates of nitrite reduction of Cygb-1, as opposed to Cygb-2, suggests that the two *D. rerio* paralogs might have acquired different biological functions after their gene duplication, with Cygb-1 exhibiting properties more consistent with an O_2_ carrier role. Based on the heme-coordination and biochemical features, zebrafish Cygb-1 may function as an ancestral respiratory globin that evolved later than the more ancient hexa-coordinated Ngb and GbX and earlier than the penta-coordinated Mb and Hb, and could perhaps represent the evolutionary connection between these two groups of respiratory globins [112,121].

## 4. NO Signaling in Teleost

Within the cardiovascular system, NO is a vasodilator promoting angiogenesis and vascular remodeling; it is protective towards tissue damage and these effects are generally mediated by the canonical NO-sGC-cGMP signaling pathway [156,157,158,159]. When released from the endothelium in response to physiologic stimuli such as shear stress, NO binds to the normally-reduced moiety of sGC and increases the formation of cGMP from GTP leading to decrease in intracellular calcium and vasodilation. At nanomolar concentrations, NO activates sGC which leads to the activation of a number of physiological processes through the activation of PKG, cGMP-cation gated channels, and cGMP-hydrolyzing phosphodiesterases (PDEs) [160]. In the vasculature, NO production and Ca^2+^ homeostasis modulate blood pressure in a dose dependent manner [4,157]. In hypertension, a reduction of NO-dependent vasodilation is partially attributed to decreased levels of sGC [161].

sGC protein is a cytosolic heterodimer protein formed by α and β subunits containing the heme moiety essential for the activation of the enzyme. NO production in fish is well-established, but only few studies have reported the direct involvement of the canonical NO-sGC-cGMP signaling pathway, and evidence of sGC-cGMP responsiveness in the fish vasculature is limited. In medaka fish, mRNA for sGC α and β subunits have been isolated and identified in retina [162], and in zebrafish, the expression of the sGC homologues *gucy1a* and *gucy1b* has been found in the adult olfactory system and are shown to be closely related to the structure of the mammalian subunits [163]. However, no functional studies have been conducted in these reports. NO is shown to have a novel pro-inflammatory role in leukocyte recruitment upon injury via sGC dependent and independent signaling, but the relevance of the NO-sGC-cGMP axis was tested limitedly by pharmacological inhibition with sGC inhibitor 1H-[1,2,4]oxadiazole-[4,3-a]quinoxalin-1-one (ODQ) [164]. Particularly of note, ODQ is a potent inhibitor of sGC but also a heme protein oxidant that can affect nitrite conversion to NO by globins proteins, thus possibly interfering with interpretations of mechanisms of NO homeostasis and signaling [165].

Irrespective of the NO generation being NOS-dependent or NOS-independent, the NO-induced effects on the cardiac response in goldfish are shown to be mediated by sGC, and its pharmacological inhibition by ODQ in goldfishes’ isolated heart induced a reduction of NO-dependent cardiac regulation [79]. In this model, the major transduction pathway is mediated by cGMP and through PKG- dependent troponin I phosphorylation results in accelerated myocardial relaxation. These effects are augmented during hypoxia and seem to contribute to the hypoxic response in the hypoxia-tolerant goldfish heart [79]. Hypoxia- and anoxia-tolerant fish well-illustrate the critical adaptive responses to O_2_ limitation by modulating NO and its metabolites. Compared to normoxic conditions, the hypoxic hearts from goldfish maintain significant levels of NO and increased NOS and HIF-1α expression that point to an important role played by the cardiac NOS/NO system as the coordinator of the cardio-protective signaling cascade ultimately improving cardiac basal performance with time [79].

Functional evidence of sGC responsiveness to NO signaling in fish has been demonstrated in *gucy1a1* zebrafish mutant larvae: blood flow and linear velocity in the main axial vessels were significantly increased at 72 h post-fertilization and the morpholino downregulation of both *gucy1a1* and *gucy1b1*-impaired cGMP production was detected in the whole homogenized larvae lysate [166]. In the skeletal muscles of zebrafish larvae, NO negatively modulates contractility through the regulation of Ca^2+^ release and uptake via direct PKG phosphorylation through the NO-sGC signaling pathway [167].

**Figure 5:**
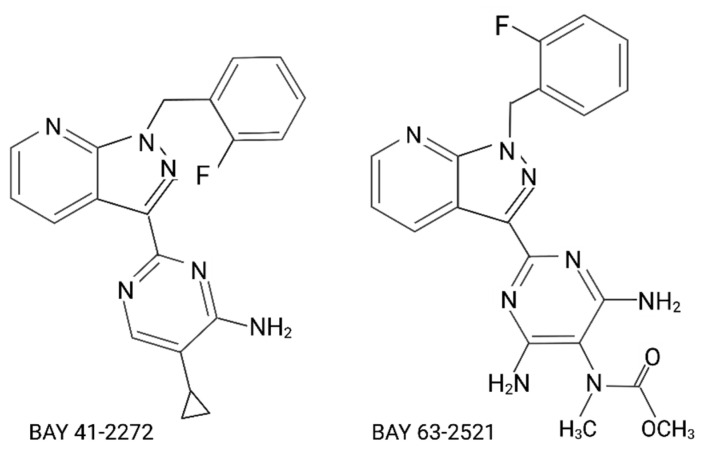
Chemical structure of selected sGC activators (BAY 41-2272 and BAY 63-2521) tested in fish.

In pathophysiologic states, both NO formation and bioavailability can be impaired by decreased NO production, oxidative stress, and if a NO tolerance has developed. Substantial efforts have been made in mammalian models to develop pharmacological regulators of sGC function in order to overcome the necessity of NO in activating the signaling pathway. Two classes of compounds have been developed that can directly activate sGC and increase cGMP formation. Both heme-dependent stimulators and heme-independent activators target the NO-sensing heme domain of the β subunit [156], although they have different mechanisms of action. Stimulators require the presence of ferrous heme-bound sGC and enhance its activity and synergize with NO activated sGC when NO is available [168,169,170]. These compounds have been extensively used in research and have been found to be effective in fish. Indeed, the modulation of sGC with stimulators can rescue the *gucy1a1* and *gucy1b*1-combined morpholino effect and restore normal levels of cGMP in the developing zebrafish larva [166], Figure 5. sGC activators are heme analogues and can trigger the activity of sGC independently from the heme-bound and its redox state [171]. These compounds have been used to stimulate oocyte maturation in zebrafish [172], but no data have been generated yet in regard to cardiovascular functions using fish models (Figure 5).

## 5. *S*-Nitrosylation of Globins

The role of NO in the control of blood flow is central in cardiovascular biology. In this regard, extensive recent evidence indicates that hypoxic vasodilation primarily includes *S*-nitrosothiol (SNO)-based vascular activity, rather than NO itself [173]. While high amounts of nitrogen species can impact cell function and induce cell death, all RNS are responsible for protein post-translational modifications, i.e., the formation of SNOs within proteins [174]. NO can also directly react with many biomolecules such as heme-containing proteins, thiols, or amines, forming iron-nitrosyl (FeNO), SNO and *N*-nitroso (NNO) compounds [19] (Figure 1), which can therefore act both as NO carriers and/or NO scavengers. Some of these biomolecules are fundamental components of the signal—transduction pathways of NO, both cGMP-dependent and cGMP-independent. Depending on cellular and tissue microenvironments, protein *S*-nitrosylation has been established as a significant route by which NO transmits its ubiquitous cellular influence [175].

In Hbs, *S*-nitrosylation is a reversible post-translational modification with an NO group covalently bound to the cysteine thiol Cysβ93 of the primary structure of the β subunit to form SNO. Jia et al. [176] were the first authors to describe the existence of a dynamic NO cycle linked to this cysteine residue with the production of *S*-nitroso Hb (SNO-Hb) in position β93. One hypothesis was that the formation of SNOs preserves and stabilizes NO bioactivity by producing bioactive, low-molecular-weight nitrosothiols to protect NO from oxidative degradation [177]. The physiological effects are relevant for mammalian Hbs where *S*-nitrosylation mediates vasodilation and vasoconstriction [178].

Tissue oxygenation is thus controlled not only by the O_2_ content, but also by the SNO content of Hb. This SNO respiratory cycle occurs in mammals, reptiles, and birds consistent with the strict evolutionary conservation of Cysβ93 [179]. In mice with a βCys93Ala mutation, tissue oxygenation and blood flow are markedly impaired, suggesting that the Cysβ93 is required for normal cardiovascular function and circulatory adaptation to hypoxia [180]. However, recently in a number of ex vivo and in vivo mice models, authors demonstrated that Cysβ93 of Hb is not necessary to mediate hypoxic vasodilation and cardioprotection [181].

In fish, the reactive cysteine residue at the position 93 of the β subunit of Hb is often replaced by a serine residue [182]. Generally, teleost fish possess one cysteine residue in the α chain and two/three cysteine residues in the β chain of Hb [179]. Then the question remains: is the SNO-respiratory control broken in fish? Scattered evidence suggests that a “respiratory *S*-nitrosylation cycle” is operative in the spot where fish (*Leiostomus xanthuurus*) have Hb possessing a βCys but not in position 93 [183]. This finding seems to indicate the importance in maintaining the SNO-cycle in vertebrates so that even if the “critical” β chain Cys93 is absent, other conformationally-reactive cysteine residues present in the molecule are recruited. Therefore, the SNO-cycle routinely demonstrated in vitro may still be found to be physiologically necessary [183].

In the heart, an excessive build-up of NO can impair cardiac functions. Mbs with reactive cysteine residues able to form SNOs in vitro are seen scattered throughout the vertebrate evolution. Human Mb has a single reactive cysteine at position 110 and is known to form SNO thiols in vitro [184,185]. In contrast to other mammalian Mbs where cysteine residues are sparse, fish Mbs most often contain one conserved cysteine residue at positions 10 of the primary structure [33,186]. Based on crystallographic analyses, it is speculated that the Cys10 residue of Mb blackfin tuna (*Thunnus atlanticus*) can form a thiol within the *N*-terminal helix that could impact the heme reactivity of the protein supposedly large enough to alter functional properties [187]. In addition to Cys10, rainbow trout and Atlantic salmon Mbs have a second cysteine residue at position 107, most likely involved in the formation of SNO. Helbo et al. recently showed that *S*-nitrosylation increases the O_2_ binding affinity of Mbs that appear to be allosterically regulated by NO [188,189]. The allosteric regulation mediated by *S*-nitrosylation was not evidenced in either yellowfin tuna (*T. albacares*) Mb or in common carp Mb1 and Mb2, where only Cys10 is present, thus suggesting that the additional Cys107 present in salmonid Mbs is the only one responsible for the observed SNO-dependent allosteric effects on O_2_ binding affinity. 

Cysteine residues are found in the Mb of most fish species sequenced thus far, suggesting that Mb may play an active role in regulating internal levels of NO in ectothermic animals, such as fish, who often experience fluctuations in tissue O_2_ availability. However, there is no evidence or functional data to support this hypothesis in vivo. Although well-described in mammals, these novel functions related to *S*-nitrosylation and thiols formation in Mb have only been examined to a very minor extent in fish so far. *S*-nitrosylation may also occur on cysteine residues of mammalian Ngb and Cygb, however their potential role in redox signaling is worthy of further investigation [190].

## 6. Conclusions

NO is a key molecule implicated in many physiological processes with roles in the nervous, cardiovascular, and immune systems [7]. Although significantly fewer studies have been published about NO metabolism and signaling in fish compared to mammals, it is now known that NO is produced in fish tissues through different pathways regulated by O_2_ concentration and can react with a variety of biological targets to mediate a number of signaling networks. The conservation of the NOS/NO system among vertebrates demonstrates the critical role played by NO and its metabolites in different fish adapted to diverse aquatic environments, from anoxic to highly oxygenated cold waters of the Southern Ocean.

In teleosts, globins play a central role in maintaining NO homeostasis, being able to catalyze reactions of NO scavenging or NO production, and by post-translational modifications capable of transducing NO signaling via non-canonical sGC-cGMP NO signaling pathways. In this context, the Antarctic icefish species are interesting examples of “naturally-occurring genetic knockout” serving as unique vertebrate models for studying the relationship between Hb/Mb and NO signaling.

Future studies are needed to reconstruct the evolutionary pathways of endothelial NO in fish and the role of globin proteins in the fish cardiovascular system.

## Figures and Tables

**Table 1:** Presence of *nos* in teleost fishes.

Species	*nos* Genes Identified	Endothelial Activity	References
*Danio rerio*	*nos1*, *nos2a*, *nos2b*	+	[59,60,78]
*Carassius auratus*	*nos1*, *nos2a*, *nos2ba*, *nos2bb*	+	[59,60,79]
*Cyprinus carpio*	*nos1*, *nos2a*, *nos2ba*, *nos2bb*	nd ^1^	[59,60]
*Oncorhynchus mykiss*	*nos1*, *nos2a*, *nos2β*	nd ^1^	[59,60]
*Salmo salar*	*nos1*, *nos2a*, *nos2β*	nd ^1^	[60]
*Piaractus mesopotamicus*	*nos1*^2^, *nos2*	nd ^1^	[66]
*Ictalurus punctatus*	*nos1*, *nos2.1*, *nos2.2*	nd ^1^	[60,67]
*Paramormyrops kingsleyae*	*nos1*, *nos2*, *nos3*	nd ^1^	[60]
*Takifugu poecilonotus*	*nos1*	nd ^1^	[59]
*Tetraodon nigroviridis*	*nos1*	nd ^1^	[59]
*Gasterosteus aculeatus*	*nos1*	nd ^1^	[59]
*Oryzias latipes*	*nos1*	nd ^1^	[59]
*Anguilla anguilla*	*nos1*	+	[74]
*Thunnus thynnus thynnus*	*nos1*	+	[74]
*Chionodraco hamatus*	*nos1*	+	[73]
*Trematomus bernacchii*	*nos1*	+	[73]
*Chaenocephalus aceratus*	*nos1*	+	[73]

^1^ Not determined. ^2^ Not identified.
